# Antispike Immunoglobulin-G (IgG) Titer Response of SARS-CoV-2 mRNA-Vaccine (BNT162b2): A Monitoring Study on Healthcare Workers

**DOI:** 10.3390/biomedicines10102402

**Published:** 2022-09-26

**Authors:** Alessio Danilo Inchingolo, Giuseppina Malcangi, Sabino Ceci, Assunta Patano, Alberto Corriero, Daniela Azzollini, Grazia Marinelli, Giovanni Coloccia, Fabio Piras, Giuseppe Barile, Vito Settanni, Antonio Mancini, Nicole De Leonardis, Grazia Garofoli, Giulia Palmieri, Ciro Gargiulo Isacco, Biagio Rapone, Megan Jones, Ioana Roxana Bordea, Gianluca Martino Tartaglia, Antonio Scarano, Felice Lorusso, Luigi Macchia, Angela Maria Vittoria Larocca, Silvio Tafuri, Giovanni Migliore, Nicola Brienza, Gianna Dipalma, Francesco Inchingolo

**Affiliations:** 1 Department of Interdisciplinary Medicine, Section of Dental Medicine, University of Bari “Aldo Moro”, 70124 Bari, Italy; 2 Department of Interdisciplinary Medicine, Intensive Care Unit Section, Aldo Moro University, 70121 Bari, Italy; 3 Department of Oral Rehabilitation, Faculty of Dentistry, Iuliu Hațieganu University of Medicine and Pharmacy, 400012 Cluj-Napoca, Romania; 4 UOC Maxillo-Facial Surgery and Dentistry, Department of Biomedical, Surgical and Dental Sciences, School of Dentistry, Fondazione IRCCS Ca Granda, Ospedale Maggiore Policlinico, University of Milan, 20100 Milan, Italy; 5 Department of Innovative Technologies in Medicine and Dentistry, University of Chieti-Pescara, 66100 Chieti, Italy; 6 Department of Emergency and Organ Transplantation (D.E.T.O.), University of Bari “Aldo Moro”, 70124 Bari, Italy; 7 Hygiene Complex Operating Unit, Azienda Ospedaliero-Universitaria Consorziale Policlinico di Bari, Place Giulio Cesare, 11, 70124 Bari, Italy; 8 Department of Biomedical Science and Human Oncology, University of Bari, 70121 Bari, Italy; 9 University Hospital of Bari, 70120 Bari, Italy

**Keywords:** MERS, SARS-CoV-2, SARS-CoV-1, COVID-19, antibodies, antispike, vaccines, dentistry, Pfizer, booster

## Abstract

The secretion of IgG SARS-CoV-2 antispike antibodies after vaccination with BNT162b2 and the protection represent the response of the human organism to the viral vector symptomatic infections. The aim of the present investigation was to evaluate the immune reaction in health workers of the Polyclinic of Bari to identify the relationship of antispike titers with blood type, sex, age, and comorbidities. This prospective observational study (RENAISSANCE) had as its primary endpoint the assessment of serologic response to BNT162b2 at three blood titers: the first at 60 days after the second dose (3 February 2021); the second titer at 75 days after the first titer; and the third titer at 130 days after the second titer. Out of 230 enrolled staff members, all responded excellently to the mRna Pfizer (BNT162b) vaccine. Only one patient, 40 days after the second dose (3 February 2021), was positive on the swab control performed on 15 March 2021, although completely asymptomatic, and was negative on the subsequent molecular swab performed on 30 March 2021. All the patients responded to the mRNA Pfizer (BNT162b) vaccine with an antispike IgG level above 500 BAU/mL at the first antispike protein essay (60 days after the second dose on 3 April 2021); at the second titer (75 days after the first titer on 20 June 2021), 4 (1.7% of 230 enrolled) patients showed an antispike IgG level under 500 BAU/mL; at the third titer (130 days after the second titer on 30 June 2021, which means 9 months after the second dose), 37 (16.1% of 230 enrolled) patients showed an antispike IgG level under 500 BAU/mL. The data analysis demonstrated that patients belonging to blood group 0, regardless of their rhesus factor, showed the strongest level of antibodies compared to the other groups. No dependency was found between low antibodies level and sex or age. Molecular swab controls were performed every 15th of the month continuously. However, the enrolled patients’ activity was at high risk because they carried out medical activities such as dental and surgical as well with droplets of water vaporized by the effect of turbines, piezosurgery. The vaccination campaign among health workers of the Policlinico of the University of Bari “Aldo Moro” led to an excellent serological response and the complete absence of COVID-19 incident cases, so the antibody response was excellent. The COVID-19 vaccine booster shot should be administered after 9 months and not without prompt antispike titer detection to assess if any sign of waning immunity is present in that specific patient.

## 1. Introduction

SARS-CoV-2 represents a viral vector with a very critical airborne transmission capability [1,2]. In fact, the air droplets’ release seems to be one of the most effective diffusion ways for COVID-19 infection [3]. Therefore, many authors indicated healthcare workers and dentists as very critical subjects for viral vector exposure due to the medical environment and the prolonged contact with potentially infected patients [4,5]. In fact, this condition seems to be associated with a confined working environment with lots of aerosol generation and risk of being infected from salivary droplets which contain the SARS-CoV-2 virus [5,6,7]. 

Many studies have demonstrated that after infection, a decline in serum anti-SARS-CoV-2 antibodies occurs, decreasing rapidly in the first 120 days after infection and then more slowly in the following 210 days while maintaining significant antibody levels for at least 11 months after infection [8,9,10,11,12]. In an analysis of 3689 adults aged ≥18 years who were admitted to 21 US hospitals in 18 states from 11 March to 15 August 2021, the efficacy values of Moderna and Pfizer-BioNTech mRNA vaccines (VE) and Jannsen vaccine were assessed. Values were estimated at 15–40 days after receiving the second dose of Moderna and Pfizer-BioNTech vaccine or the single dose of Janssen vaccine [13]. The EV levels were 93% for Moderna and 88% for Pfizer, respectively, while the single dose Janssen vaccine had a slightly lower EV of 71%. These results suggest that the double-dose protection of the mRNA vaccines (Pfizer-BioNTech and Moderna) is greater than the 1-dose Janssen. Moderna vaccine showed an efficacy of 93% at 2–17 weeks (median = 66 days) after receiving the second dose of vaccine and 92% at >17 weeks (median = 141 days) (*p* = 1.000). In contrast, the Pfizer-BioNTech vaccine showed a significantly reduced VE of 91% (median = 69 days) and 77% (median = 143 days), respectively. Moderna also produced higher levels of post-vaccination anti-RBD antibodies than the Pfizer-BioNTech vaccine. The VE of the Moderna vaccine is better than Pfizer-BioNTech vaccines, because the mRNA content in the Moderna vaccine is greater, as are the time intervals between doses (28 days for Moderna and 21 days for Pfizer) [14,15]. However, in this study, variant-specific VE, including Delta variants (B.1.617.2 and AY underlines), were not assessed. In another study of 3975 healthcare personnel, 204 (5%) were positive for SARS-CoV-2, of which 5 were vaccinated ≥2 weeks after the second dose, 11 were partially vaccinated ≥2 weeks after the first dose and <2 weeks after the second dose, and 156 were not vaccinated. Persons (5%) became infected. Vaccine efficacy was 91% with full vaccination and 81% with partial vaccination. In addition, in partially or fully vaccinated infected subjects, the mean viral RNA load was 40% lower than in unvaccinated subjects, as well as 58% fewer febrile symptoms and a shorter illness of 2.3 days [14]. The VE of the mRNA vaccines towards the Delta variant were reduced, and this was higher than in the Pfizer vaccine (Moderna 76%, Pfizer 42%) [16,17]. In conclusion, however, the data ensure that all COVID-19 vaccines approved or self-released by the FDA ensured substantial defense against hospitalization for COVID-19 at 99% [18]. The association of the improved antibody response linked to the longer time interval between the first and second administration (see Moderna/Pfizer) can be correlated to the concept of the binding energy or receptor affinity that a B-cell has for a given antigen. This is the same affinity as the antibodies secreted by the same B-cell after antigenic stimulation. Thus, with the attendance of small doses of antigen, cells with high-affinity receptors will be stimulated more, resulting in the secretion of high-affinity antibodies. Conversely, larger doses of antigen will induce lower affinity antibodies. This relationship between immunogen dose and antibody affinity explains why, as time passes after immunization and the concentration of antigen in the body decreases, antibody affinity increases. Since it is mainly B cells with high affinity receptors that are stimulated, the average affinity of antibodies in the serum increases. The attendance of antibodies aids this selection by competing with cellular receptors for the antigen. During the secondary response, the increased affinity of the antibodies may be because of the stimulation of B cells with high affinity receptors, which occurred during the primary response when antigen concentrations were progressively decreasing over time. It is hypothesized that the secondary response would result from the stimulation of cells qualitatively different from those of the primary response [17,19,20,21,22,23,24,25]. The role of the booster dose of the primary cycle is to achieve a greater immune response and to ensure a good level of defense against infection [26]. The main goal of immunization during the COVID-19 pandemic is to improve the clinical course by avoiding hospitalization and reducing mortality. Therefore, the third dose should only be done if it is clear that there is no protection against these disease outcomes of the disease over time. According to the CDC (Center for Disease Control and Prevention) recipients of the COVID-19 vaccine who can get booster shots (Pfizer-BioNTech or Moderna COVID-19 vaccine) are [27]:

- Elderly 65 years of age or older: people aged 65 and over should receive a booster injection. The risk of severe COVID-19 disease increases with age.
- Long-term care facility residents aged 18 and over: Long-term care facility residents live closely together in group settings.
- People with comorbidities between the ages of 18 and 64.
- People who work or live in high-risk environments between the ages of 18 and 64.

The FDA and CDC suggest a booster dose at least 4 weeks after the second dose of Moderna or Pfizer, or 60 days after the first dose of Janssen/Johnson & Johnson for people who have comorbidities associated with immunosuppression [28,29,30,31,32]. On 30 July 2021, Israel became the first to give a booster dose of Pfizer against COVID-19 to all persons aged over 60 years who had been immunized at least 150 days previously. At 2 weeks after the booster dose, there was an 11.4-fold chance of infection and a >10-fold lower chance of severe disease. Against the Delta variant, the efficacy of Pfizer’s third dose was about 95%, a similar value to the efficacy of the original vaccine, which had been reduced from 85% to 75% against severe forms [33]. The EMA is evaluating the use of the third dose for Moderna. Data from the pharmaceutical company’s studies have shown significant anti-pal responses 15 days after the third dose of the Moderna vaccine (a 50 microgram booster dose of mRNA-1273): more than 40-fold against the Delta variant (B.1.617.2), 32-fold against Beta (B.1.351), and 43.6-fold against Gamma (P.1). In addition, the reactogenicity of a third BNT162b2 mRNA COVID-19 vaccine was analyzed. A study conducted on seniors and immunocompromised individuals reported that local and systemic side effects were analogue to those who received prior doses. [34] Bensouna et al. analyzed the humoral immunity after a booster dose of Pfizer in 69 persons cured with either hemodialysis or peritoneal dialysis. In this analysis, the third dose was performed at least four weeks after the second dose. Results showed a substantial rise in the antibody level in the sample. However, there was not a significant rise in the antibody level after a booster dose in persons who were undergoing chemotherapy or in those with initial high anti-S1 antibodies [35]. The target of the present investigation was to assess the short and long term immune profiling vaccine response in healthcare workers.

## 2. Materials and Methods 

### 2.1. General Characteristics

Since March 2020, the staff involved in patient care has undergone periodic screening with molecular tests for SARS-CoV-2 infection at the university hospital consortium polyclinic of Bari. Subsequently, the vaccination campaign with the vaccine BNT162b2 mRNA COVID-19 (Pfizer, New York, NY, USA) began at the same institution. For the purposes of this analysis, hospital staff were asked to assess antibody dynamics after vaccination. In this analysis, 230 healthcare workers from different departments were included (90 dentistry, 72 radiology, 34 forensic medicine, 34 internal medicine) of which 23 operators contracted SARS -CoV-2. The health care groups evaluated belong to 4 areas:

- dental area: dental physicians, chair assistants, hygienists, and nurses: a total of 90 were evaluated. (39.13% of 230 total)
- radiological area: radiology physicians, technicians, and nurses: a total of 72 were evaluated (31.30% of a total of 230)
- internal medicine area: a total of 34 were evaluated (14.78% of a total of 230);
- Forensic Medicine area: a total of 34 (14.78% of 230 total) were evaluated.

In order to estimate the antibody titer decay the sample population was categorized into 4 different classes of age range:

- Group I: subjects between 20–30 years old;
- Group II: subjects between 30–40 years old;
- Group III: subjects between 40–50 years old;
- Group IV: subjects between 50–60 years old;
- Group V: subjects between 60–70 years old;

The antibody levels of the recruited healthcare workers were evaluated. Overall, the antibody response was assessed circa ten months after vaccination. Anti-SARS-CoV-2 Spike IgG antibodies were assessed with the LIAISON^®^ SARS-CoV-2 TrimericS IgG assay (DiaSorin, Saluggia, Italy), which can show both binding and neutralizing antibodies to the trimeric Spike glycoprotein. Subjects were engaged from 11 January 2021 (first dose) and 3 February 2021 (second dose). The occurrence of vaccine-associated viral infections was assessed by RT-PCR on symptomatic/contact cases through 30 September 2021. All health care workers enrolled in the research were given a nasopharyngeal swab every 15th of each month to assess the onset of COVID-19 after the second vaccine. All enrolled in the research always performed swabs every 15th of the month starting in May 2020. Therefore, the enrolled healthcare workers were screened and did not contract SARS-CoV-2 already from 15 May 2020. The last sampling for assessing the antibody levels was carried out 270 days after the first dose of the Pfizer vaccine. The subjects enrolled in the analysis were monitored with molecular swab on the 15th of each month and none were positive. Thus, the registered healthcare professionals had all been screened and certainly did not contract SARS-CoV-2.

### 2.2. Statistical Analysis

The sample size calculation was performed considering an effect size f^2^: 0.05; α error: 0.05 and power (1-β): 0.80, and 3 predictors. The determined population output was 222 subjects which was increased by 4% as an eventual drop-out compensation.

Descriptive statistical analysis was conducted by the program Microsoft Excel (Microsoft, Redmond, WA, USA) by calculating the average, max, and min level of the antibody titers for different groups of patients by blood type, number of vaccination shots, titers test titers, gender and age, as well as the correlation coefficients by test titers. The normal distribution of the study data was assessed through the Kolmogorov–Smirnov test. The Kruskal–Wallis followed by the Dunn’s post-hoc test was conducted to evaluate the mean differences of the study groups. 

The level of significance was *p* < 0.05.

## 3. Results

**Figure 1:**
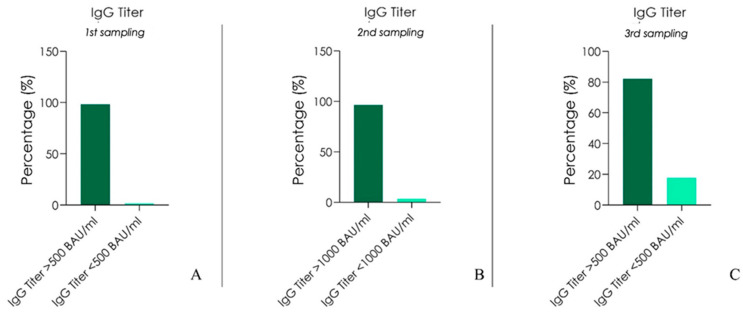
(**A**): Percentage of enrolled persons at the first titer below and above the 1000 BAU/mL threshold of antispike IgG. (**B**): Percentage of enrolled persons at the second titer below and above the 500 BAU/mL threshold of antispike IgG. (**C**): Percentage of enrolled persons at the third titer below and above the 500 BAU/mL threshold of antispike IgG.

All the patients responded to the mRNA Pfizer (BNT162b) vaccine with an antispike IgG level above 500 BAU/mL at the first antispike protein Essay (2 months after the second dose on 3 April 2021): 100% of personnel had anti-S IgG titers ≥2000 BAU/mL, 19.2% between 1500–2000 BAU/mL, 9.8% between 1000–1500 BAU/mL, and 3.4% ≤1000 BAU/mL (Figure 1A). 

**Figure 2:**
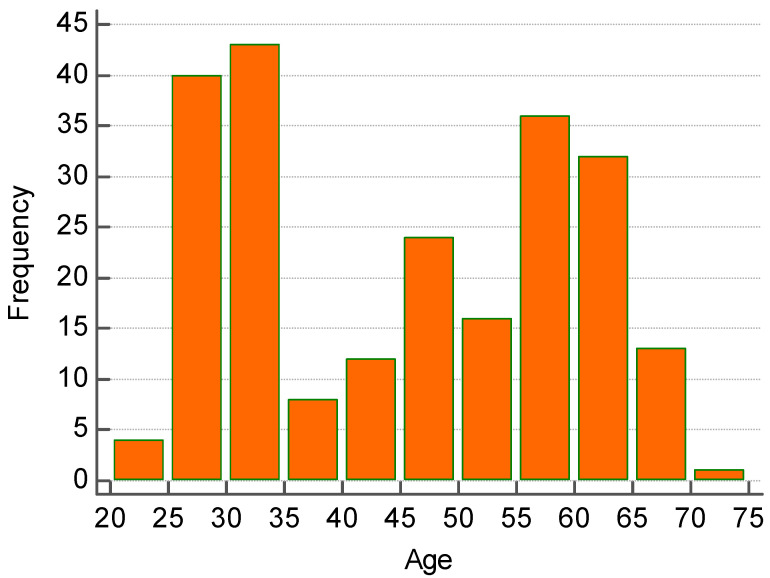
Age distribution of enrolled patients.

At the second titer (75 days after the first titer on 20 June 2021) 4 (1.7% of 230 enrolled) patients showed antispike IgG level under 500 BAU/mL. (Figure 1B). At the third titer (130 days after the second titer on 30 June 2021, which means 9 months after the second dose) 37 (16.1% of 230 enrolled) patients showed antispike IgG level under 500 BAU/mL; this percentage adds to the 1.7% of the second titer for a total 17.8% that fell below the above threshold. (Figure 1C). Seven months after the conclusion of the vaccination program, only one subject (0.43% of 230 enrolled) had SARS-CoV-2 infection, but without any symptoms and negativization after 15 days. Our descriptive analysis (Figure 1 and Figure 2 points to the fact that patients belonging to blood group 0, regardless of their rhesus factor, showed the strongest titer of antibodies compared to group A, B, and AB in each of the three titers (Figure 1 and Table 1). Age range of the analysis was the following (Figure 2):

1. Between the age of 20 and 30 years old there were 45 subjects (19.57% of 230 enrolled).
2. Between the age of 30 and 40 years old there were 52 subjects (22.61% of 230 enrolled)
3. Between the age of 40 and 50 years old there were 34 subjects (14.78% of 230 enrolled).
4. Between the age 50 and 60 years old there were 53 subjects (23.04% of 230 enrolled)
5. Between the age of 60 and 70 years of age there were 46 subjects (20% of 230 enrolled).

**Figure 3:**
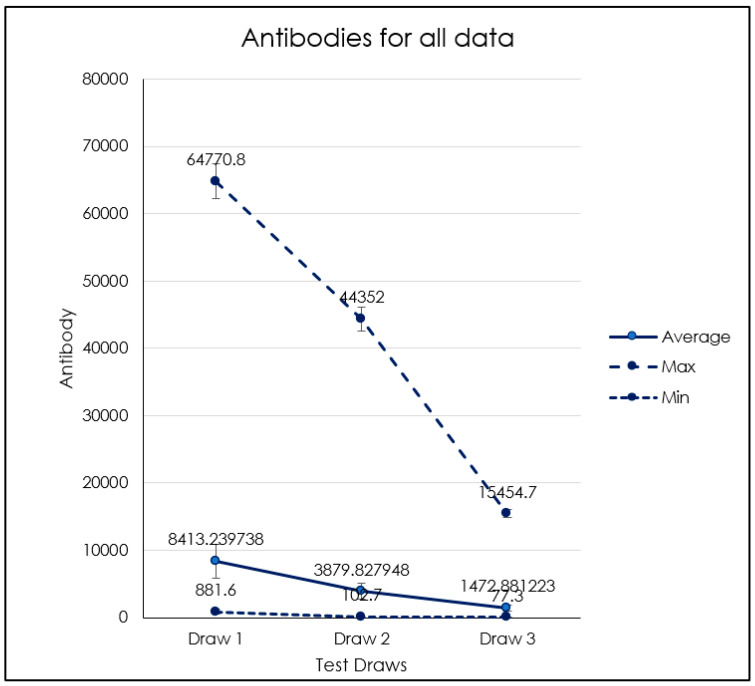
Average, max, and min titers of antibodies for the entire sample.

**Figure 4:**
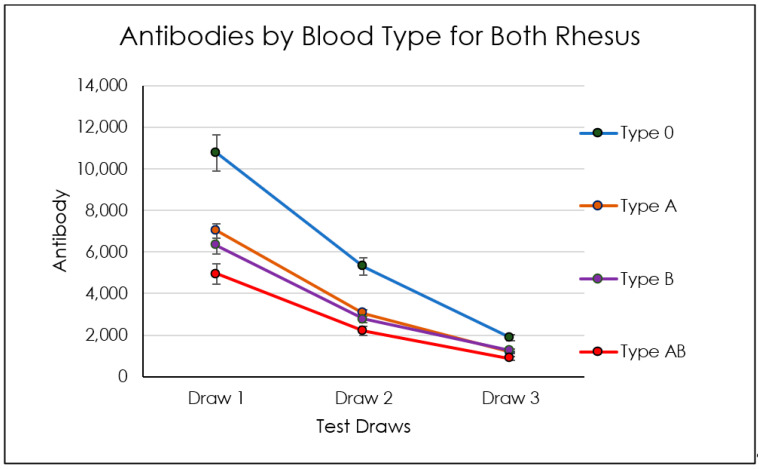
Average, max, and min titers of antibodies by blood type regardless of rhesus factor.

**Figure 5:**
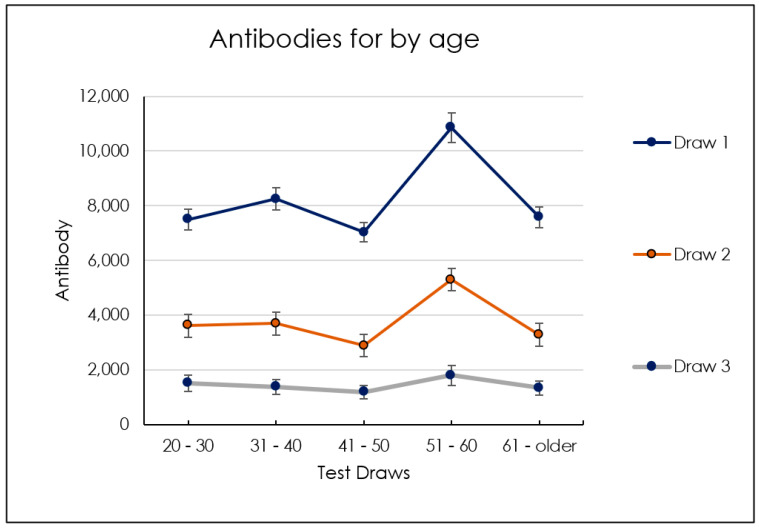
Dynamics of average levels of antibodies by age.

**Figure 6:**
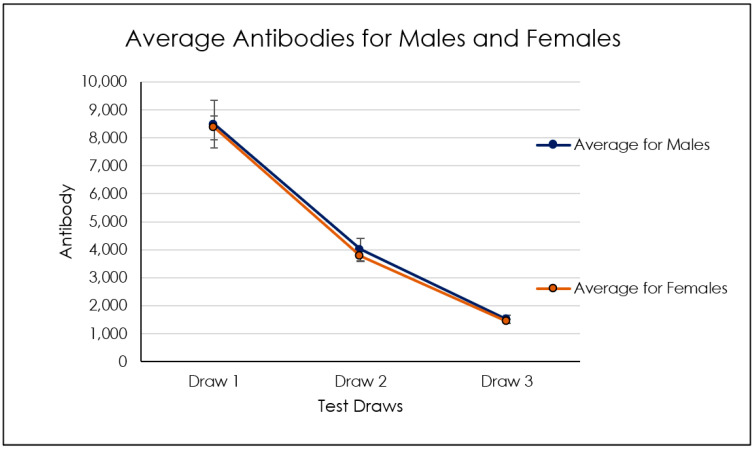
Average levels of antibodies for males and females.

**Figure 7:**
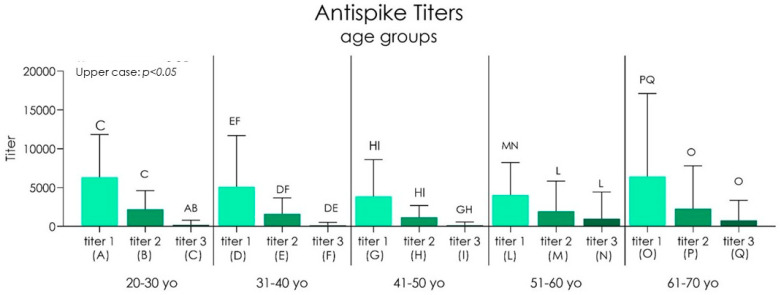
Chart of the antispike IgG level referred to all age groups.

In the age group between 50 and 60, we detected an increase of antibody levels in all three titers compared to the other groups, which showed instead approximately close average antibody levels between the different titers (Figure 3, Figure 4, Figure 5, Figure 6 and Table 1). No dependency with the antibodies level was found on gender (Figure 7, Table 2 and Table 3).

### 3.1. Statistical Findings

#### 3.1.1. Age-Related Findings

**Figure 8:**
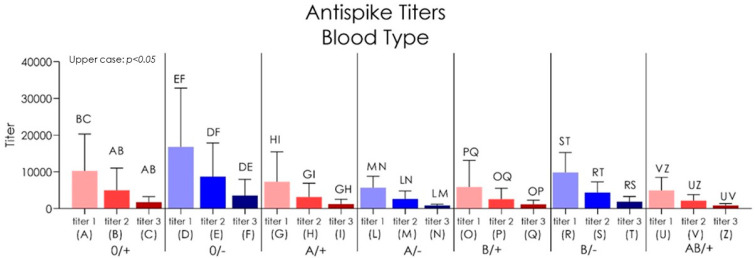
Chart of the antispike IgG level referred to all blood type groups.

Group I showed antispike IgG level means and standard deviations of 6342 ± 5506 BAU/mL at titer 1, 2207 ± 2397 BAU/mL at titer 2, and 207.6 ± 599.8 BAU/mL at titer 3 (Table 4). The antispike IgG level of group II reported a 5118 ± 6593 BAU/mL at titer 1, 1628 ± 2041 BAU/mL at titer 2, and 151.3 ± 377.0 BAU/mL at titer 3. Group III showed 3871 ± 4737 BAU/mL at titer 1, 1172 ± 1522 BAU/mL at titer 2, and 169.0 ± 414.3 BAU/mL at titer 3. Group IV reported an antispike IgG level of 4046 ± 4174 BAU/mL at titer 1, 1963 ± 3872 BAU/mL at titer 2, and 1010 ± 3413 BAU/mL at titer 3. Group V showed 6438 ± 10,573 BAU/mL at titer 1, 2289 ± 5513 BAU/mL at titer 2, and 780.6 ± 2578 BAU/mL at titer 3 (Table 4). A significant difference was detected between groups I, II, IV, and V between the antispike IgG level at titer 1 (*p* < 0.05), 2, and 3, while a lower antispike IgG decrease was detected between the titer 1 and 2 of group III (*p* > 0.05) (Figure 8). The comparison of antispike IgG level titer 1, 2, and 3 showed no statistically significant differences between all age groups (*p* < 0.06) (Figure 7).

#### 3.1.2. Blood-Type-Related Findings

Group 0/+ type showed antispike IgG level means and standard deviations of 10,289± 10,013 BAU/mL at titer 1, 5025 ± 6024 BAU/mL at titer 2, and 1739 ± 15.48 BAU/mL at titer 3 (Figure 8; Table 5). The antispike IgG level of 0/− type group reported a 16,810 ± 15,992 BAU/mL at titer 1, 8710 ± 9160 BAU/mL at titer 2, and 3561 ± 4414 BAU/mL at titer 3. The A/+ group III showed 7327 ± 8160 BAU/mL at titer 1, 3159 ± 3748 BAU/mL at titer 2, and 1246 ± 1291 BAU/mL at titer 3. Group A/− type reported an antispike IgG level of 5717 ± 3095 BAU/mL at titer 1, 2666 ± 2138 BAU/mL at titer 2, and 872.8 ± 333.2 BAU/mL at titer 3. The B/+ blood type group showed 5867± 7293 BAU/mL at titer 1, 2574 ± 2965 BAU/mL at titer 2, and 1167 ± 1142 BAU/mL at titer 3 (Table 5). The antispike IgG level of the B/− type group reported 9862 ± 5421 BAU/mL at titer 1, 4387 ± 2881 BAU/mL at titer 2, and 1873 ± 1435 BAU/mL at titer 3. The antispike IgG level of the AB/+ type group reported 4945 ± 3577 BAU/mL at titer 1, 2193 ± 1625 BAU/mL at titer 2, and 876.8 ± 397.4 BAU/mL at titer 3. A significant difference has been detected between the antispike IgG level comparing the titer 1, 2, and 3 for all blood type groups (*p* < 0.05). The stratified comparison of the antispike IgG titer level showed no statistically significant differences between all blood type groups (*p* < 0.05).

## 4. Discussion

The population sampling of the present investigation was conducted in order to include according to a more equal distribution healthcare workers from a medical/surgical interventional area and doctors from a non-interventional medical area. The differences of healthcare work exposure could produce a sensible critical point in the population enrollment and a potential limit of the study. On the contrary, this approach is able to produce a more consistent sample size and consequently a higher statistical power.

Immunological memory is a property of both T and B lymphocytes [4,36,37,38,39]. In an antiviral response, cytotoxic T lymphocytes selectively eliminate the infected cells; neutralizing antibodies secreted by plasma cells preventing the virus from infecting them [37,40,41,42,43,44,45,46,47,48,49,50,51,52]. Virus-specific T helper cells are required to generate immunological memory, particularly for long-lasting bone marrow plasma cells (BMPC), which secrete antiviral antibodies when the virus has disappeared for long-lasting immunity [28,36,53,54,55,56,57,58,59,60,61]. The bone marrow (BM) is one of the main immunological organs in the human body. BMPCs are detected in the BM and in gut-associated lymphoid tissues (GALT), which produce antibodies for a lot of time [40,62]. Studying the serum values of patients convalescing from COVID-19 at 1, 4, 7, and 11 months, it was detected that infection with SARS-CoV-2 provokes a transient and early response with a high production of extrafollicular (spleen and lymph nodes) antibodies, which decrease relatively quickly [63,64,65,66]. Subsequently, more stable serum antibodies secreted by long-lasting BMPC are detected. In fact, analysis of bone marrow aspirates obtained approximately 7 and 11 months post-infection revealed S-specific anti-SARS-CoV-2 BMPC [67,68,69,70]. Consequently, circulating anti-S IgG titers at 210–240 days after symptom begin in convalescent individuals is related with the concentration of anti-S IgG BMPC present in the bone marrow aspirate [40,62]. All convalescent subjects who received a dose of mRNA vaccine increased all components of the humoral reaction. The data confirm that BMPC expressing specific antibodies are long-lasting, have serum neutralizing activity against new variants of concern, and are cleared and produced extensively after vaccination. These data suggest that immunity in convalescent persons will be very long-lasting. Individuals who contracted COVID-19 and received mRNA vaccines will produce antibodies and memory B cells that will also be protective against circulating SARS-CoV-2 variants [71,72,73,74,75]. Research at the Washington University School of Medicine in St Louis, Missouri, on the value of the memory B-cell response, analyzed fragments from the lymph nodes of vaccinated patients and found ‘germinal centres’, i.e., tiny areas of B-cell refinement, which, over time, synthesized increasingly powerful immune cells, thus being able, through this evolutionary process, to fight the Delta variant and other worrying SARS-CoV-2 variants. The persistence of these germinal centers was detected at 15 weeks post immunization [13].

Our study discovered that after 270 days after the second dose, most of the enrolled patients still showed a significant antispike titer. This is in contrast with what Yinon M. Bar-On et al. showed in their analysis [76]. We think that antispike titers greater than 500 BAU/mL can still deliver protection as it should be noted that a decline in serum antibodies does not mean that there is a lowering of immunity but rather a rising of it with the development and persistence of SARS-CoV-2 memory CD8^+^ T cells, SARS-CoV-2 memory B Cells and SARS-CoV-2 memory CD4+ T cells in the bone marrow [77]. According to the age variable, no significant differences were detected between the the study groups at titers 1, 2, and 3 (*p* > 0.05). In fact, the groups seemed to produce similar fluctuations and a consistent decrease in the antispike IgG levels. Similar evidence was detected according to the he blood type groups that only the O negative blood groups seemed to produce a more consistent level of antispike IgG (*p* < 0.05) at titer 1 and titer 2. No differences in titer 3 were detected between the blood types in the present investigation (*p* > 0.05). This correlation with COVID-19 protection activity has been suggested by several authors but the association is not completely cleared and is controversial according to the current literature. In addition, very few data are reported in relation to the vaccination effectiveness. Rana et al. reported on a single center study that the A, B, and Rh+ blood groups were susceptible to COVID-19 infection in comparison to blood groups O and Rh− [78]. Very similar findings were reported on different populations groups such as household and children [79].

Certain subsets of individuals might also carry some form of protection having a much higher antibody’s titer compared to other subsets: in our study, we demonstrated that patients belonging to group zero may have this “enhanced” protection due to a higher antispike titer that lasts longer over time. The subset of patients aged 50–60 might have this increase in antibodies’ level because of some undetected comorbidity that in our hypothesis could lead to this immunological picture. Any other study at the moment does not support this finding. No dependency with the antibodies’ level was found with gender. This is in contrast with what Shachor-Meyouhas et al. [80] showed in their analysis where the male sex was identified as a risk factor for lower antibody level in an observational timeline of 3 months after the second shot. The strength of our analysis is that it extended over 9 months after the second shot. The COVID-19 vaccine booster shot should therefore be administered after 9 months and not without prompt antispike titer detection to assess if any sign of waning immunity is present in that specific patient. It must be hence noted that the majority of the subjects enrolled in our study were protected against COVID-19 even after 9 months after the first dose of the vaccine despite their activity being a high risk because they carried out medical, dental, and surgical activities, and with droplets of water vaporized by the effect of turbines, piezosurgery. More studies are required to assess waning immunity kinetics in specific subsets of persons with specific traits such as comorbidities and other anamnestic data.

In healthy adults, two 30 μg doses of BNT162b2 elicited high neutralizing titers and robust, antigen-specific CD4^+^ and CD8^+^ T cell responses against SARS-CoV-2 [57,58]. Therefore, it revealed 95% efficacy among phase 2–3 study subjects aged 16 years or older [57]. Although BNT162b2 is a two-dose regimen, early protection after a single dose has been reported in clinical trials and based on real data [59,60]. A significant titer decay has been detected by Israel et al. [81] in a preliminary report on a wide population screening on BNT162b2 vaccinated subjects, reporting different antibody kinetics between vaccinated patients and convalescents. At 6 months after vaccination the 16.1% patients reported an antibody titer below the seropositivity threshold of <50 AU/mL, while 10.8% convalescent subjects were below <50 AU/mL 9 months after COVID-19 infection. A high titer of autoimmune antibodies in COVID-19 patients has been registered, although it is not clear how these antibodies help in the progression of the disease and its clinical picture. These antibodies were studied in a retrospective study of 115 hospitalized COVID-19 patients who had different clinical manifestations; the reaction of autoimmune antibodies to common antigens such as erythrocyte lysate, lipid phosphatidylserine (PS), and DNA was tested. In up to 36% of patients, a large quantity of IgG autoantibodies against erythrocyte lysate was detected.

Anti-DNA and anti-PS antibodies recorded when the patients were admitted to hospital showed an interconnection with the severity of the disease: the positive predictive values were 85.7 and 92.8, respectively. Persons with good values for at least one of the two autoantibodies were rated at 24% of the total severe cases. Recent studies reveal that coagulation, neutrophil levels, markers of cell damage, and erythrocyte size are strongly correlated with anti-DNA antibodies. Anti-DNA and anti-PS autoantibodies can potentially be considered predictive biomarkers in the typology of a clinical course of COVID-19. Long COVIDs are those who present with the persistence of symptoms or the development of new symptoms related to SARS-CoV-2 infection, at least 28 days after diagnosis. Symptoms may be constant or intermittent and may be multi-organ [82]. Dyspnea, tachycardia, and extreme tiredness are more frequent despite the normalization of the inflammatory parameters. Negative RT-PCR diagnostic test values, undoubtedly related to fibrosis induced by cytokine storms in the acute phase, led to chronic pulmonary and cardiac damage, with reduced flow in spirometry tests, high titers of troponin T (TnT), and brain natriuretic peptide (BNP). There are values thought to be because of fibrosis remodeling with transforming growth factor (TGF)-beta secretion in the chronic phase, with overlapping results in other diagnostic tests (ultrasound or chest CT) [83,84,85]. Symptoms include night sweats, temperature changes, gastrointestinal tract disorders [86], constipation/soft stools and peripheral vasoconstriction due to autonomic nervous system dysfunction [87]. In a study at the Policlinico Universitario di Bari, the characteristics and risk factors of 35-day long COVID (35-LC) were investigated over one year from 8 March 2020 to 15 March 2021. The analysis assessed the age, gender, and symptom characteristics of the first week. A distinction was made between persons with a short course of infection (less than 10 days) (<10 days COVID) and those who had been symptomatic for at least 28 days (28 days COVID or 28-LC). Adverse outcomes were not shown to be localized. Instead, they were present in several systems, including the immune system (e.g., Guillain-Barré syndrome, rheumatoid arthritis, pediatric multisystem inflammatory syndromes, such as Kawasaki disease), and the hematological system (vascular hemostasis), depression and anxiety and a condition called ‘brain fog’, which causes difficulties in attention and concentration. Molecular mechanisms associated with these disease outcomes/symptoms have been correlated [88]. Under well-being conditions, the host’s microbiome/virome [89] ecosystem is held in check by an effective host immune defense and persists in a state of equilibrium or homeostasis. In fact, dysbiosis leads to dozens of chronic metabolic changes [90]. Microbiome/virome dysbiosis may favor the growth of opportunistic pathogens. Immune dysregulation induced by SARS-CoV-2 may lead to an imbalance in the body’s existing microbial and viral ecosystems that may cause long-term multi-thyroid functional alterations with multiple symptoms [91]. In fact, almost all organisms in human microbiome/virome communities are ‘pathobionts’, namely they are able to change their gene pool to become pathogenic organisms under conditions of unbalance and immunosuppression [92]. It is also possible that, after becoming infected, SARS-CoV-2 persists in certain parts of the body or tissues in some persons, causing chronic symptoms [93,94,95].

## 5. Conclusions

The present study findings seems to suggest no differences of the different variables evaluated among the selected population groups. Blood groups A, B, and Rh+ seem to produce a similar response to the vaccination treatment with similar trends in a medium-short follow up. Blood groups O− seem to indicate an higher antispike IgG titer medium-short terms that could potentially support the higher protection against the SARS-CoV-2 infection. Therefore, long term studies with a larger sample size are needed to assess the relationship of between blood groups and the response to the SARS-CoV-2 vaccines.

## Figures and Tables

**Table 1:** Results for all blood types.

	All Blood Types		
	Titer 1	Titer 2	Titer 3
Average	8.413	3.880	1.473
St.Dev	9.510	5.156	1.818
Max	64.771	44.352	15.455
Min	882	103	77
Range:	63.889	44.249	15.377
# Patients		229	
	Titer 1,2	Titer 2,3	Titer 1–3
Correlation	0.97	0.80	0.81

**Table 2:** Results for all ages.

	Ages Related Blood Types		
	Titer 1	Titer 2	Titer 3
Average	8.413	3.880	1.473
St.Dev	9.510	5.156	1.818
Max	64.771	44.352	15.455
Min	882	103	77
Range:	63.889	44.249	15.377
# Patients		229	
	Titer 1,2	Titer 2,3	Titer 1–3
Correlation	0.97	0.80	0.81

**Table 3:** Blood types referred to all genders.

	Genders Referred Blood Types		
	Titer 1	Titer 2	Titer 3
Average	8.413	3.880	1.473
St.Dev	9.510	5.156	1.818
Max	64.771	44.352	15.455
Min	882	103	77
Range:	63.889	44.249	15.377
# Patients		229	
	Titer 1,2	Titer 2,3	Titer 1–3
Correlation	0.97	0.80	0.81

**Table 4:** Antispike IgG level referred to all age groups.

	Group I 20–30 yo			Group II 31–40 yo			Group III 41–50 yo			Group IV 51–60 yo			Group V 61–70 yo		
	titer 1	titer 2	titer 3	titer 1	titer 2	titer 3	titer 1	titer 2	titer 3	titer 1	titer 2	titer 3	titer 1	titer 2	titer 3
Mean	6342	2207	207.6	5118	1628	151.3	3871	1172	169.0	4046	1963	1010	6438	2289	780.6
SD	5506	2397	599.8	6593	2041	377.0	4737	1522	414.3	4174	3872	3413	10673	5513	2578
Lower 95% CI	4668	1479	25.29	3264	1054	45.30	2243	657.3	28.83	2896	895.2	69.02	3269	670.2	23.75
Upper 95% CI	8016	2936	390.0	6972	2202	257.4	5498	1687	309.2	5197	3030	1951	9608	3907	1537

**Table 5:** Antispike IgG level referred to the blood type groups.

	0/+			0/−		
	Titer 1	Titer 2	Titer 3	Titer 1	Titer 2	Titer 3
Mean	10,289	5025	1739	16,810	8710	3561
SD	10,013	6024	1548	15,992	9160	4414
Lower 95% CI of mean	8001	3648	1385	27.84	903	−1071
Upper 95% CI	12,577	6401	2093	33,593	18,322	8192
	**A/+**			**A/−**		
	Titer 1	Titer 1	Titer 1	Titer 1	Titer 2	Titer 3
Mean	7327	5717	5717	5717	8710	3561
SD	8160	3095	3095	3095	9160	4414
Lower 95% CI of mean	5008	3638	3638	3638	903	−1071
Upper 95% CI	9646	7797	7797	7797	18,322	8192
	**B/+**			**B/−**		
	Titer 1	Titer 2	Titer 1	Titer 2	Titer 1	Titer 2
Mean	5867	2574	5867	2574	5867	2574
SD	7293	2965	7293	2965	7293	2965
Lower 95% CI of mean	2856	1350	2856	1350	2856	1350
Upper 95% CI	8877	3798	8877	3798	8877	3798
	**AB/+**					
	Titer 1	Titer 1	Titer 1			
Mean	4945	4945	4945			
SD	3577	3577	3577			
Lower 95% CI of mean	2672	2672	2672			
Upper 95% CI	7218	7218	7218			

## Data Availability

All experimental data to support the findings of this study are available upon request by contacting the corresponding author. The authors have annotated the entire data-building process and empirical techniques presented in the paper.

## Abbreviations

ACE2	angiotensin-converting enzyme-2
ACE	angiotensin-converting enzyme
ACE1	angiotensin-converting enzyme 1
AIFA	Agenzia Italiana del Farmaco
Alfa	English variant B.1.1.7
anti-RBD IgG	Immunogloublin G anti receptor-binding domain
Antispike	Test IgG Antispike
BAU	unità arbitrarie vincolanti
Beta variant	(former of South Africa)
BMI	Body mass index
CI	Interval of confidence
CLIAs	chemiluminescence immunoassay
CRP	C-reactive protein
Delta	Indian variant B.1.617.2
ELISA	enzyme-linked immunosorbent assay
EMA	European Medicines Agency
ETA	variant B.1.525; Date of designation March 2021
Gamma	Brasilian variant P.1
hACE2 receptor	human angiotensin I-converting enzyme 2 receptor
IFN	Interferon
IgA	Immunoglobulins A
IgG	Immunoglobulins G
IgM	Immunoglobulins M
IOTA	variant B.1.526; earliest documented samples USA (November 2020), Date of designation March 2021
IQR	Interquartile range
KAPPA	Indian variant B.1.617.1
LAMBA	variant C.37; earliest documented samples Peru (August 2020), Date of designation June 2021
LFIAs	lateral flow immunoassays.
MERS	Middle East Respiratory Syndrome
MMF	mycophenolate mofetil
MPA	mycophenolic acid
MPPDH	inosine-5’-monophosphate dehydrogenase
NAAT	nucleic acid amplification test
NGS	Next Generation Sequencing
bNAbs	Broadly neutralizing antibodies
N-IgG	Anti-N-IgG
PRD	Viral Prion-like domain
RBD	receptor-binding domain
RBDs	receptor-binding domains
RDB-IgG	receptor-binding domain neutralizing antibodies
RT-PCR	real-time PCR Polymerase chain reaction
S	the Spike glycoprotein
SARS-CoV-1	Severe Acute Respiratory Syndrome Coronavirus 1
SARS-CoV-2	Severe Acute Respiratory Syndrome Coronavirus 2 (COVID-19)
SARSr-CoV Rp3	salivar protein similar to fused 8a and 8b SARS-CoV Beta Coronavirus
S-IgG	Antispike IgG
thio-NAD	thionicotinamide-adenine dinucleotide
TNF	Tumor Necrosis Factor
VIPIT	prothrombotic immune thrombocytopenia
VOC	Variants of Concern
VOI	Variants of Interest
ZETA	variant P.2
